# NeuTHOR Station—A Novel Integrated Platform for Monitoring BNCT Clinical Treatment, Animal and Cell Irradiation Study at THOR

**DOI:** 10.3390/life13030800

**Published:** 2023-03-15

**Authors:** Yu-Shiang Huang, Jinn-Jer Peir, Chuan-Jen Wu, Mei-Ya Wang, Yi-Wei Chen, Jia-Cheng Lee, Fong-In Chou

**Affiliations:** 1 Nuclear Science and Technology Development Center, National Tsing Hua University, Hsinchu 300044, Taiwan; 2 Department of Heavy Particles and Radiation Oncology, Taipei Veterans General Hospital, Taipei 11217, Taiwan; 3 Department of Medical Imaging and Radiological Technology, Yuanpei University of Medical Technology, Hsinchu 30015, Taiwan; 4 Institute of Nuclear Engineering and Science, National Tsing Hua University, Hsinchu 300044, Taiwan

**Keywords:** NeuTHOR Station, integrated platform, BNCT, THOR

## Abstract

(1) Background: A well-established Boron Neutron Capture Therapy (BNCT) facility includes many essential systems, which are the epithermal neutron beam system, on-line monitoring system (OMS), QA/QC (quality assurance or quality control) system, boron concentration (BC) measurement system, and treatment planning system (TPS). Accurate data transmission, monitoring, and deposition among these systems are of vital importance before, during, and after clinical, animal, and cell BNCT irradiation. This work developed a novel integrated platform NeuTHOR Station (NeuTHORS) for BNCT at Tsing Hua Open-pool Reactor (THOR). Apart from the data of the OMS and QA/QC system, the data of BC and TPS can be loaded on NeuTHORS before BNCT clinical, animal, and cell irradiation. (2) Methods: A multi-paradigm computer programming language c# (c sharp) was used to develop the integrated platform NeuTHORS. The design of NeuTHORS is based on the standard procedures of BNCT treatment or experiment at THOR. Moreover, parallel testing with OMS-BNCT (the former OMS) and QA/QC of THOR was also performed for more than 70 times to verify the validation of NeuTHORS. (3) Results: According to the comparisons of the output, NeuTHORS and OMS-BNCT and QA/QC of THOR show very good consistency. NeuTHORS is now installed on an industrial PC (IPC) and successfully performs the monitoring of BNCT Treatment at THOR. Patients’ f BC and TPS data are also input into NeuTHORS and stored on IPC through an internal network from BC measurement room and TPS physicist. Therefore, the treatment data of each patient can be instantaneously established after each BNCT treatment for further study on BNCT. NeuTHORS can also be applied on data acquisition for a BNCT-related study, especially for animal or cell irradiation experiments. (4) Conclusions: A novel integrated platform NeuTHOR Station for monitoring BNCT clinical treatment and animal and cell irradiation study has been successfully established at THOR. With this platform, BNCT radiobiology investigations will be efficiently performed and a thorough data storage and analysis system of BNCT treatments or experiments can thus be systematically built up for the further investigation of BNCT at THOR.

## 1. Introduction

**Figure 1:**
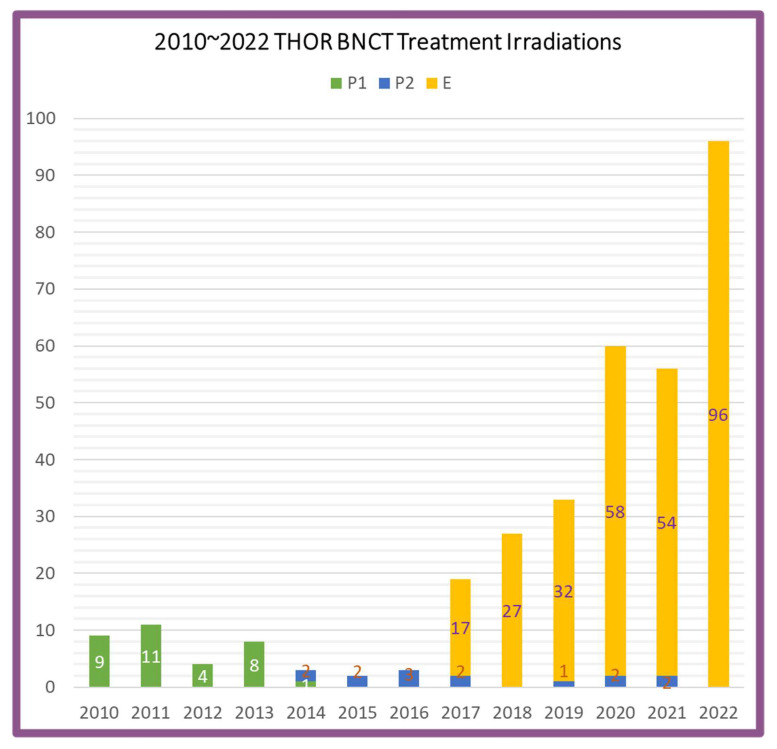
The BNCT treatment irradiations performed at THOR from 2010 to 2022. The notations P1, P2 and E indicate protocol 1, protocol 2, and emergent (compassionate) treatment, respectively.

Boron Neutron Capture Therapy (BNCT) epithermal beam irradiation at Tsing Hua Open-pool Reactor (THOR), a 2-MW rated power research reactor activated in 1961, started its commissioning after the completion of BNCT facility’s construction in 2005 [1]. Since then, the attention of many scientists and researchers of different specialties was drawn to BNCT for related investigations, such as animal or cell irradiation, at National Tsing Hua University (NTHU). In addition, the BNCT facility at THOR later successfully moved to the preclinical study and clinical trials. It has performed two clinical trials for recurrent head and neck cancer and 284 salvage treatments for mainly recurrent head and neck cancer, malignant brain tumor, or other indications before the end of December 2022. To date, 250 patients were treated at the facility, and among them, 70 patients received multi-irradiation, and 331 irradiation sessions (including 177 brain tumors, 151 head and neck cancers, and 3 other cancers) have been executed in total for BNCT treatment at THOR from 11 August 2010 to 28 December 2022. Figure 1 demonstrates the statistics of BNCT treatment irradiation times and the BNCT treatment irradiations performed at THOR during the years 2010–2022. As can be seen in Figure 1, the treatment irradiations increase quickly with the salvage BNCT of various cancers introduced after 2017. Moreover, more than 200 treatment irradiations were accomplished between 2020 and 2022. All patients are treated by the doctors from Taipei Veterans General Hospital (TVGH). Notably, the BNCT facility of THOR is presently the only reactor-based BNCT facility worldwide that is used for clinical purposes that has been treating patients in the past decade [2] and is available to the BNCT community all over the world [1,2,3,4]. Apart from Taiwan, patients are currently from Australia, Mainland China, Japan, Singapore, Brazil, Spain, Italy, and USA. The numerous treatment activities have gathered interest and stimulate many scientists and researchers to conduct further approaches in BNCT animal and cell studies at NTHU or other universities in Taiwan. As a result, in the last recent three years, the accumulated radiobiology data on BNCT clinical treatment and animal and cell irradiation have been rapidly increasing at THOR BNCT facility. 

The THOR BNCT facility includes many essential systems, such as the epithermal neutron beam system, on-line monitoring system (OMS) [5,6,7], QA/QC (quality assurance or quality control) system, boron concentration (BC) measurement system, and treatment planning system (TPS). The neutrons of THOR BNCT are monitored by a LabView based PC-program OMS-BNCT that was previously developed in 2006 [7,8]. Recently, a lot of BNCT irradiations for head and neck cancer and brain tumor clinical treatment [9,10,11,12,13,14,15,16,17,18,19], animal models of hepatoma study [20,21,22,23,24,25], studies targeting specific agents [26,27], or cell study of hepatocellular carcinoma, liver cancer, and melanoma [28,29,30] have been carried out at THOR. To upgrade the data acquisition capability and system hardware of the BNCT facility of to meet the requirements of users, an advanced platform with on-line monitoring, precise transmission, high storage rate, and data analysis ability is crucial. It will make BNCT radiobiology investigations before, during, and after clinical, animal, and cell irradiation more efficient. Especially for BNCT clinical treatment, the synchronization of relevant irradiation data can highly promote the collaboration of medical groups to achieve the quality objectives of therapy. In this work, we aimed to develop a novel integrated platform named NeuTHOR Station (NeuTHORS) with functions of OMS, QA/QC, parameter input from BC and TPS systems for dose prescription or recording before BNCT clinical, animal, and cell irradiation, and data analysis for further BNCT study at THOR.

## 2. Materials and Methods

At the THOR BNCT facility, there are three neutron beam monitoring detectors that are Centronic FC4A miniature fission chambers (FC) with identification names Chamber-A, Chamber-B, and Chamber-C; these were assembled and mounted inside the BSA (beam shaping assembly) of the epithermal neutron beam [8]. The individual measurement channel includes an FC, a charge-sensitive preamplifier, an amplifier, a single-channel analyzer, and a high voltage supply. The logic pulses output from the BNCT detector system are counted by the former system software OMS-BNCT based on LabView virtual instrumentation [7]. 

**Figure 2:**
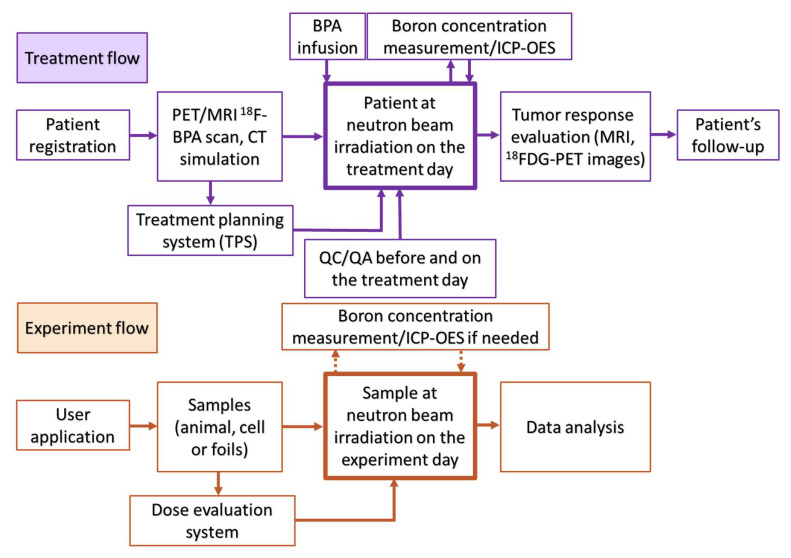
The standard procedures for BNCT treatment and experiment.

**Figure 3:**
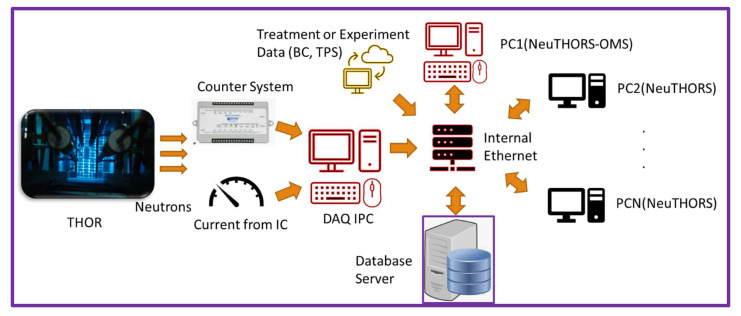
The system configuration of NeuTHORS.

In this work, a digital I/O USB data acquisition device (ADVANTECH USB-4751L-AE) was used to replace the former NI (National Instruments) plugged-in counter board on PC for counting neutrons. The main integrated platform NeuTHORS was installed on an IPC (industrial PC) with ADVANTECH ASMB-786G2-00A1 server board, i7 CPU, 4 DDR4 2666 16GB memory, and ethernet. A multi-paradigm computer programming language c# (c sharp) built into the integrated development environment (IDE) visual studio 2019 released by Microsoft was used to develop the NeuTHORS. The design of NeuTHORS is based on the procedures of BNCT treatment or experiment at THOR (Figure 2). There are two sub-flows for BNCT application: treatment flow and experiment flow. After registration or application and with the completion of corresponding data input, the operator can use NeuTHORS to monitor the irradiation status of the reactor control system. The treatment or experiment data is saved in a database server for further query, analysis, and study. Figure 3 shows the logical flow of NeuTHORS for BNCT monitoring. As can be seen, the configuration includes THOR, epithermal neutrons from the core, a counter system, ion chamber (IC) current of QA/QC, DAQ (Data Acquisition) IPC, treatment, or experiment data input by wireless or ethernet system, PCs of operators, and ethernet system. An internal wireless or ethernet connection was chosen for the purpose of data security, given that it is patient information that is being collected.

According to the current BNCT treatment procedures at THOR, there are key parameters, namely blood BC, organ target dose, background dose rate, and boron dose rate, that must be input into the OMS-BNCT for the prescribed dose evaluated by Tsing Hua- BNCTplan (TH-BNCTplan), THORplan, SERA (Simulation Environment for Radiotherapy Applications), or other TPS before the patient’s irradiation [1,31,32,33]. Moreover, at 1 h and 2 h after the initial administration of the boronophenylalanine (BPA) infusion, the first and second blood samples were taken for BC measurement by using PerkinElmer’s Avio 200 ICP-OES (Inductively Coupled Plasma Optical Emission Spectrometer). The BC values of each blood sample were measured three times, and the average BC was used for the prescription dose and final dose evaluation. In OMS-BNCT, parameter handouts were individually provided by the BC measurement group and TPS group and could only be manually keyed in by the staff members in the BNCT control room. In NeuTHORS, these parameters can not only be manually input by the BC measurement group but also directly imported from the data file of the TPS group. Moreover, we found that for many patients who had undergone phlebotomy, the time needed to start irradiation during THOR BNCT treatment was longer. The irradiation may need to be started before the BC value is fed back to the operator to avoid running out of time to administer the BPA drug. The dynamically change function of BC and irradiation rate evaluated by TPS is included in NeuTHORS even during irradiation. Therefore, apart from the data report function, the on-line change and pause functions of TPS and BC parameters can also be included for the dynamic data of each patient, if needed. Any THOR operator can use NeuTHORS-OMS, but only a superuser can be the controller and avoid any interruptions during irradiation. Moreover, multiple users can co-work simultaneously by PCs through the internal network even when working with different aspects of THOR.

**Figure 4:**
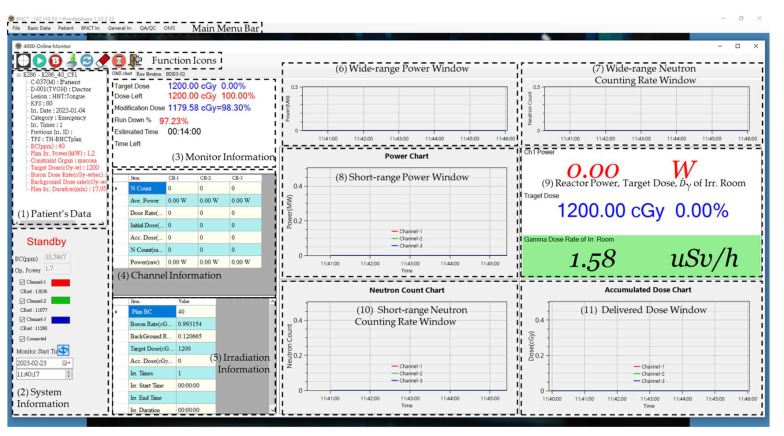
The main GUI menu and OMS dialog of the NeuTHORS at the standby stage. Additional dialog boxes have been developed for the whole structure.

The database server stores patients’ data, neutron counting rates (raw data and with dead time calibration), IC current of QA/QC, TPS, BC, and dose data. The graphical user interface (GUI) main menu bar of NeuTHORS includes the following functions: files for administration of users and the system settings, basic data establishment for the treatment or experiment, patient data for treatment irradiation, BNCT treatment-related data for BNCT irradiation, normal irradiation for register, QA/QC for ion chamber data monitoring, and OMS for the BNCT usage and chamber query for any research study. Figure 4 shows the main GUI menu and OMS dialog of NeuTHORS at the standby stage. The functions of NeuTHORS consist of several dialogs. Additional dialog boxes were developed for the individual monitoring operation. Among them are the four dialog boxes that will mainly be used; they are: (1) BNCT irradiation, for data input of clinical treatment, animal, and cell experiments; (2) Normal irradiation, for the user application of general irradiation at THOR; (3) QA/QC, for the BNCT QA/QC testing before the patient’s BNCT irradiation; and (4) OMS, for the real-time monitoring of neutrons, power, gamma dose in the irradiation room, and prescribed dose of the BNCT irradiation and QA/QC current. To verify the validation of NeuTHORS, the parallel testing of neutrons and the prescribed dose is executed at the same time with OMS-BNCT. The NeuTHORS-OMS can be quickly operated using the function icons shown in Figure 4 to select the irradiation TPS, start and stop the record, show the BC data from measurement, load the OMS-BNCT data, reset to the default scale of charts, erase standby jobs, open a sharing popup window, and leave the NeuTHORS-OMS.

In the NeuTHORS-OMS treatment flow, there are 11 blocks, as can also be seen in Figure 4. They are: (1) patient’s data; (2) system information; (3) monitor information; (4) channel information; (5) irradiation information; (6) wide-range power window (beginning from the starting time); (7) wide-range neutron counting rate window (beginning from the starting time); (8) short-range power window (in the most recent 5 min); (9) reactor power, target dose, and gamma dose rate of the irradiation room; (10) short-range neutron counting rate window (in the most recent 5 min); and (11) delivered dose window. Block (1) presents the basic data of the treatment patient, which includes the patient’s information input from the application form and the irradiation dose rate from the TPS. Block (2) shows the system information, including the running status bar, the BC from the BC measurement room, the operation power, the FC4A reference counting rate at the THOR power 1.2 MW, and the startup time of NeuTHORS-OMS. Block (3) displays the monitor information, namely the planning dose constrain, the remaining prescription dose, the corrected prescription dose, the percentage of reactor run down, the elapsed irradiation time, and the remaining irradiation time. The BNCT supervisor and operators will mainly refer to this monitor information block to manage the irradiation procedures. Block (4)~(11) demonstrate the key operation parameters, neutron counting rates, reactor power, and accumulated dose with tables, charts or digit numbers for the control of the prescription dose. The auto-scaling function was set for the irradiation parameter charts to enable the automatic display scaling. BNCT irradiation at THOR requires a close collaboration between the supervisor, the operators, the doctor, and the technicians. With NeuTHORS-OMS, they can all access the information and thus complete the work smoothly. 

Furthermore, NeuTHORS-OMS can also be utilized in the experiment flow following the treatment flow mentioned above; however, this is without the patient’s data and measured BC of patient. The animal or cell BC can be input for the BNCT-related experiment if the irradiation dose evaluation value was estimated or measured in advance. Hence, the operator can operate the THOR irradiation session according to the objective dose of the samples instead of the operation time, which is occasionally too short to control. Moreover, this skill operation can eliminate the control deviation that occurs from different operators being involved. The physical target dose or weighted target dose of the animal or cell can also be calculated following a similar evaluation method of patients by TPS or dose evaluation program. Then, the parameters of blood BC, organ target dose, background dose rate, and boron dose rate of the animal or cell can be input into the NeuTHORS for the precise control of the BNCT irradiation dose. Thus, it will elevate the quantity control of the experiment with regards to the dose. NeuTHORS-OMS can also be used for animal or cell experiments upon an application request from research users.

To validate the dose accuracy and delivery quality to patients, quick QA/QC checks of the beam will be performed twice before the BNCT treatment irradiation at the THOR BNCT facility. One is usually performed a day before the treatment date and the other is on the day of the BNCT treatment irradiation. The QA/QC of the BNCT beam combines activation foils and paired ionization chamber techniques [7]. The neutron counting rates of the three FC4A channels with dead time calibration can be depicted by the following equation:

(1) $${CR}_{meas}=A\times {CR}_{correct}+B\times {{CR}_{correct}}^{2}$$

where ${CR}_{correct}$ denotes the corrected FC4A counting rate, A the dead-time correction parameter, B the dead-time correction parameter, and ${CR}_{meas}$ the measured FC4A counting rate. From Equation (1), ${CR}_{correct}(t)$ can be further solved as

(2) $${CR}_{correct}(t)=\frac{-A+\sqrt{A^{2}+4\times B\times {CR}_{meas}(t)}}{2B}$$

The above dead-time correction equation will be embedded in NeuTHORS as a function to restore the raw counting rates. The corrected counting rates and the measured FC4A counting rate will both be exported on the OMS for cross referencing. The corrected counting rates will be used as the prescribed dose evaluation of BNCT. Moreover, the FC4A reference counting rate obtained from the activation foils (Au and Cu) can be deduced by

(3) $${CR}_{ref}=\frac{{RR}_{ref}}{A}\sum_{t=1}^{T}CR\left(t\right){(1-e^{-\lambda })e}^{-\lambda \left(T-t\right)}$$

where ${CR}_{ref}$ is the FC4A reference counting rate at the THOR power 1.2 MW, ${RR}_{ref}$ the Au/Cu foil reference/theoretical reaction rate, A the Au/Cu foil measured radioactivity, $CR\left(t\right)$ the FC4A counting rate during irradiation time T, λ the Au/Cu foil decay constant, and T the Au/Cu foil irradiation time. 

Moreover, the total target dose of patients evaluated from TPS can be calculated by the following equation:

(4) $$D_{total}=\sum_{t}^{}\left[{\dot{D}}_{b10}\times \frac{{BC}_{icpoes}}{{BC}_{plan}}+{\dot{D}}_{other}\right]\times \frac{{CR}_{correct}\left(t\right)}{{CR}_{ref}}$$

where $D_{total}$ is the total target dose evaluated from TPS, ${\dot{D}}_{b10}$ the boron dose rate obtained from TPS, ${\dot{D}}_{other}$ the background dose rate obtained from TPS, ${BC}_{plan}$ the boron concentration predicted from TPS, and ${BC}_{icpoes}$ the boron concentration obtained from ICP-OEMS. Likewise, the users can also follow the treatment procedures for the dose evaluation of animal or cell irradiation by using TPS or the dose evaluation program to obtain $D_{total}$, ${\dot{D}}_{b10}$, and ${\dot{D}}_{other}$ in advance and then using NeuTHORS-OMS to perform the reactor operation and monitor the irradiation experiment. The reference irradiation time of the animal or cell experiment is calculated by NeuTHORS after the setup of $D_{total}$, ${\dot{D}}_{b10}$, and ${\dot{D}}_{other}$, and the operator can get all of the synchronization information through a screen to control the BNCT irradiation. The relevant irradiation data can be exported to the users after the completion of the experiment.

In addition to the data acquisition and display of the neutron beam counting rates, data query function, which exports data in CSV format, is also included in NeuTHORS to help the further analysis and research study of any specific case in the future. The reactor power level is also displayed on the NeuTHORS to help the BNCT supervisor and operator control the overall conditions of the THOR-BNCT operation. Moreover, during BNCT treatment, a popup window appears with the following key parameters: the prescribed dose, the percentage of prescribed dose, the power level of THOR, the elapsed time and remaining time of irradiation, and the BC of the patient. This is shared from the BNCT control room to the doctor who is sitting in front of the patient’s surveillance camera, so that they have access to the treatment information. Authentication ability with user approval and authorization function is designed for the management of THOR. Daily backup is performed every day at midnight for server data protection.

## 3. Results and Discussion

**Figure 5:**
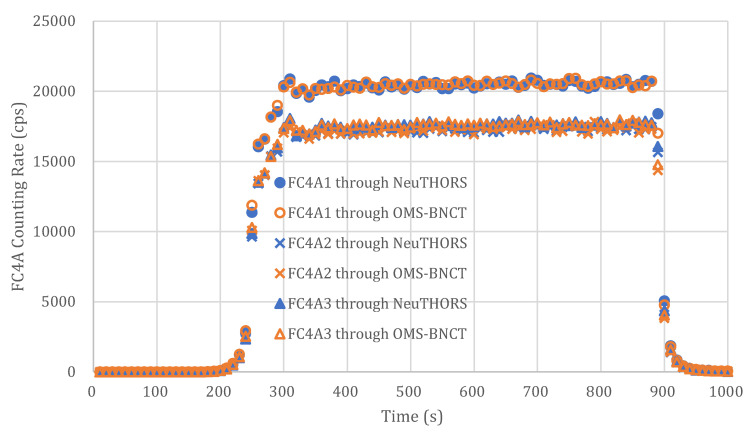
Beam monitor counting rates with dead time correction of a BNCT irradiation session recorded by the NeuTHORS and OMS-BNCT.

**Figure 6:**
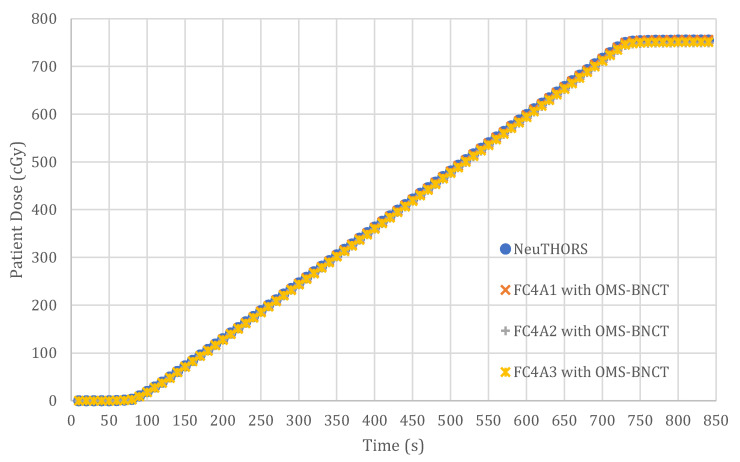
The prescribed dose of a BNCT irradiation session evaluated by the NeuTHORS and OMS-BNCT.

NeuTHORS has been developed and applied to run on the Windows platform at THOR BNCT control room. The corresponding measurements of neutrons and the evaluation of the prescribed dose of NeuTHORS-OMS and OMS-BNCT are performed at the same time to allow for comparisons and verification. Figure 5 presents the measured counting rates of a BNCT irradiation session simultaneously recorded by NeuTHORS and OMS-BNCT. Moreover, Figure 6 shows the prescribed dose of a BNCT irradiation session evaluated by the NeuTHORS and OMS-BNCT. According to the comparisons of the output of counting rates and prescribed dose, the results of NeuTHORS and OMS-BNCT show very good consistency. As can be seen in Figure 5 and Figure 6, at the very beginning, THOR’s start-up to irradiation power (usually 1.7 MW) takes about four minutes. Once THOR reaches a higher power (above 20 kW), the beam line neutrons gradually increase. When the neutron counts increases to more than 100, the prescribed dose starts to register till the target dose has been achieved. At this time, the BNCT supervisor informs the reactor operator of the control room to switch THOR off. Then, the irradiation treatment is complete.

**Figure 7:**
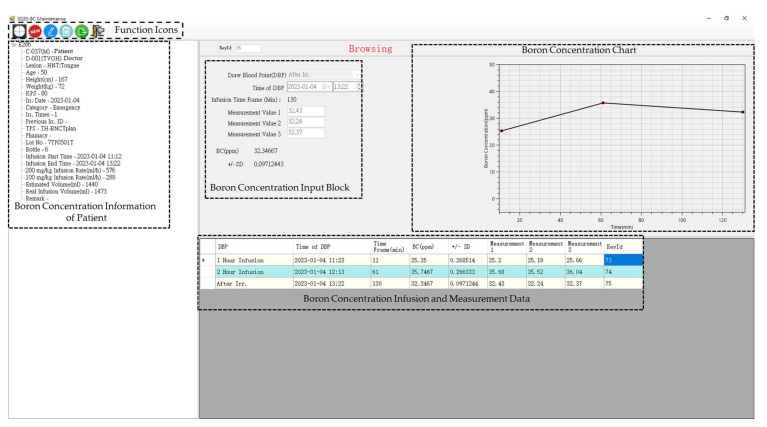
The boron concentration dialog of a salvage patient measured by ICP-OES input from the measurement group through NeuTHORS during treatment irradiation.

Figure 7 describes the blood BC of a salvage patient measured by ICP-OES input from the BC measurement group through NeuTHORS during the treatment irradiation. After the BPA injection, three patient blood samples at one hour, two hours, and after the irradiation, are individually taken and measured by the BC measurement group and transmitted to the supervisor and operator in the BNCT control room for the dose prescription and final dose evaluation. Three BC measurements are done for each sample. The data of measured BC is keyed in and all is stored on the IPC server. The mean of these three BC values is worked out and the correlated chart is then plotted accordingly by NeuTHORS. When the measurement results of the blood BC at 2-h injection is decided, the supervisor and operator in BNCT control room then set the BC, the irradiation parameters prepared and imported from TPS, and the power level of THOR. Following the step-by-step confirmation, which includes ensuring the patient’s correct alignment and positioning, the installation of a mobile camera for the patient’s surveillance, the closure of the beam shielding door, and the double-checking of the irradiation parameters, the BNCT treatment irradiation is started. 

**Figure 8:**
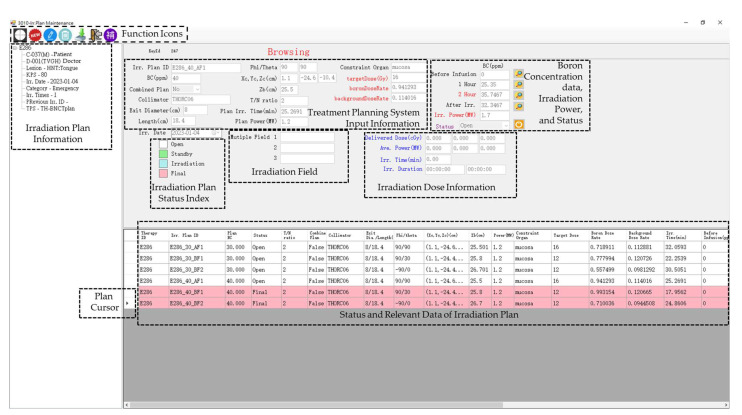
The irradiation treatment plan dialog of a salvage patient in NeuTHORS. Six preliminary treatment plans were imported from treatment planning system Tsing Hua- BNCTplan. Plan BF1 and BF2 with 40 ppm boron concentration were selected as the irradiation plans in this case. After the accomplishment of BNCT treatment irradiations, the pink bars show up and the operation status is changed from irradiation to final. The white bars indicate that the irradiation plans are in open status.

Figure 8 is the irradiation treatment plan dialog of a salvage irradiation performed by NeuTHORS. There are six preliminary treatment plans designed by the TPS physicist using TH-BNCTplan in this case. As shown, there are one-field treatment plans AF1 with 30 ppm and 40 ppm BC and two-field treatment plans BF1 and BF2 with 30 ppm and 40 ppm BC imported from TH-BNCTplan to NeuTHORS. After detailed discussions and comparisons of plan A and plan B, the two-field treatment plans B were selected by the medical doctor as the irradiation plans. The average blood BC of the patient was 35.7467 ppm at the two-hour injection and the irradiation power was set to 1.7 MW. Due to the BC exceeding 35 ppm, plan BF1 and plan BF2 with 40 ppm boron concentration were therefore chosen as the irradiation plans in this case. After the accomplishment of BNCT treatment irradiations, the pink bars show up and the status is changed from “irradiation” to “final”. The white bars indicate that the plans are in open status. The open status of plan AF1 with 30 ppm and plan AF1 with 40 ppm, plan BF1 with 30 ppm and plan BF2 with 30 ppm BC indicate that the plans were not being used in this case. 

**Figure 9:**
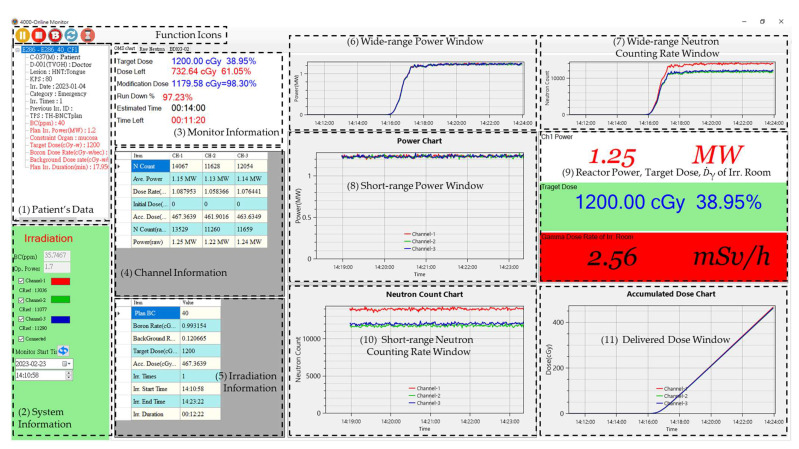
The NeuTHORS-OMS ongoing display of a salvage BNCT at irradiation stage.

Figure 9 shows the NeuTHORS-OMS ongoing display of a BNCT treatment irradiation session performed recently. When the arrangement is ready for BNCT treatment irradiation, the NeuTHORS-OMS can be started by pressing a virtual button (the second icon on the NeuTHORS-OMS function icon bar) shown on the screen with a left mouse click. After that, only the selection icon of the irradiation TPS, record icon of the stop, BC icon export from BC measurement room, reset icon of the default scale of charts, and popup icon of the sharing window are shown on the function icon bar. As can be seen in Figure 4 and Figure 9, the status bar in block (2) is changed from shut down mode to in operation mode and the BNCT-related parameters are shown in block (3)~(11). Moreover, in block (3), the monitor information of BNCT irradiation, including planning dose constrain, the remaining prescription dose, the corrected prescription dose, the percentage of reactor run down, the elapsed irradiation time, and the remaining irradiation time appears on the monitor. The BNCT supervisor and operators can synchronously watch those parameters in the BNCT control room and THOR control room to control the irradiation. Individual channel information can be obtained from block (4). Irradiation information is summarized in block (5). Furthermore, five chart windows and one digit number window displaying the irradiation data on the right of the NeuTHORS-OMS also appear. Block (6) and block (8) show the wide-range power chart and the short-range power chart, respectively. Block (7) and block (10) present the wide-range neutron counting rate and short-range neutron counting rate, respectively. Block (9) shows the reactor power, target dose, and gamma dose rate of the irradiation room in digit numbers, and block (11) illustrates the accumulated dose chart during the BNCT irradiation. The short-range blocks present the data curves in the latest five-minute interval. The wide-range blocks show the data curves after the NeuTHORS startup. In OMS-BNCT, only two charts are presented on the screen; one is the accumulated prescription dose and the other is the neutron counting rate. Moreover, the neutron counting rate block only shows the data curves within the most recent two minutes and without the corresponding reactor power indication on the screen. Thus, this multi-block display mode is a noteworthy improvement for monitoring purposes and help the BNCT supervisor and operators obtain the regional and global irradiation information needed to set the correct operation conditions.

**Figure 10:**
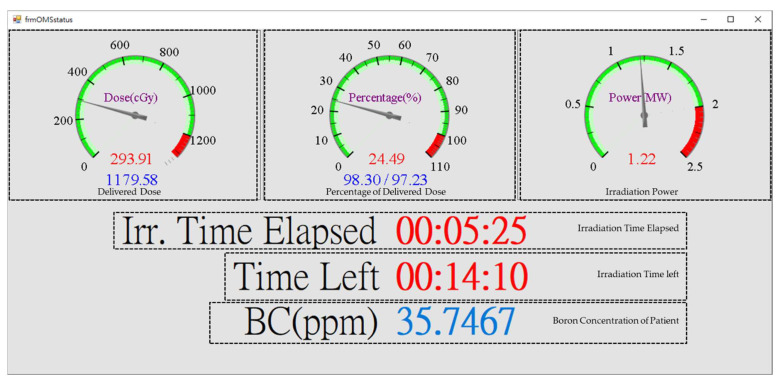
The popup monitoring window of the NeuTHORS-OMS that a doctor sees during the salvage BNCT irradiation of a patient.

The BNCT supervisor in the BNCT control room and the operators in the THOR control room can mostly examine the monitor information block to communicate with each other and oversee the irradiation condition. The automatic display scaling of the irradiation parameter charts of neutron counting rates, reactor power, and accumulated dose promotes cooperation between the supervisor, the operators, and the BNCT treatment team technicians on BNCT irradiation. Figure 10 illustrates the corresponding popup monitoring window of the NeuTHORS-OMS sharing to the attending physician during BNCT irradiation treatment. As shown in Figure 10, it is a panel-like window mixed with digit numbers that show the key irradiation information of the BNCT irradiation treatment. The attending physician and accompanying family of the patient can obtain the prescribed dose, the percentage of the prescribed dose, the power level of THOR, the elapsed time and the remaining time of irradiation, and the BC of the patient from the sharing window. They can use this practical information, in particular the remaining time of irradiation, to support the patient during the irradiation treatment.

**Figure 11:**
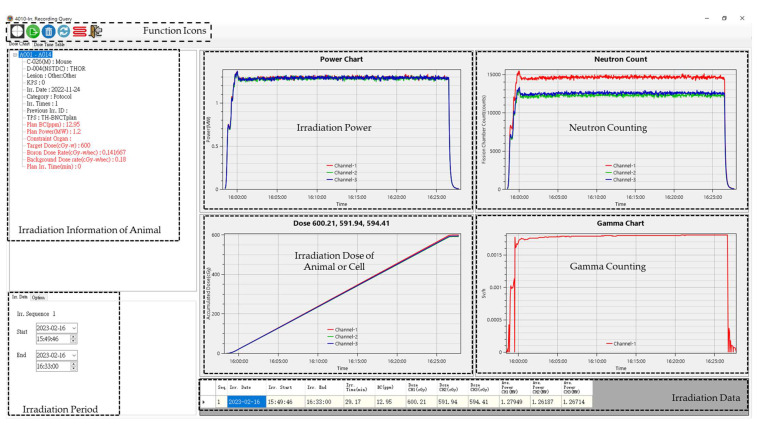
The NeuTHORS-OMS recording of an animal experiment after BNCT irradiation at THOR.

NeuTHORS is now installed on an industrial PC (IPC) and has successfully performed the monitoring of BNCT treatment and cell experiments at THOR more than 70 times. The patient’s BC and TPS data from the BC measurement room and TPS physicist are also input into the NeuTHORS and stored on IPC through the internal network. Therefore, the treatment data of each patient can be instantaneously established after each BNCT treatment for further study on BNCT. NeuTHORS can also be applied on the data acquisition on BNCT-related studies, especially for animal or cell irradiation experiments. Given that the irradiation parameters can similarly be obtained by TPS or program evaluation, a BNCT animal and cell irradiation study can simply follow the treatment procedures to monitor the irradiation and obtain the required data immediately after the experiment. Figure 11 is the NeuTHORS-OMS recording of a mouse experiment after BNCT irradiation. As can be seen from the irradiation information of the animal and the irradiation data blocks, the boron concentration of the mouse is 12.95 ppm, the estimated target dose is 600 cGy-w, the boron dose rate is 0.1417 cGy-w/s, and the background dose rate is 0.18 cGy-w/s. All the former parameters were provided by the users and input into NeuTHORS by a THOR researcher. The irradiation time was originally estimated to be 30 min at 1.2 MW, whereas the real operation time was 29.17 min at an average power of 1.28 MW, as shown on Channel-1. The real irradiation dose shown on Channel-1 is 600.21 cGy, which is less than 0.1% of the target dose. By using NeuTHORS-OMS, the irradiation time and dose can be significantly controlled and the irradiation deviation of the BNCT experiment can be reduced. Further function extensions of NeuTHORS according to the requirements of users can be performed quickly and easily due to the flexibility and compatibility of the C sharp system. The data loading performance of NeuTHORS is very smooth and efficient in relation to its low disk capacity; about 0.5 M bytes is needed, even with daily files stored on the server.

## 4. Conclusions

A novel integrated platform NeuTHORS for monitoring BNCT clinical treatment, animal and cell irradiation study was successfully established at THOR in this work. With this platform, BNCT radiobiology investigation will be efficiently performed and a thorough data storage and analysis system of BNCT treatments or experiments can thus be systematically built up for the further investigation of BNCT at THOR. A function extension of NeuTHORS can be carried out upon the requirements of the users. Expanding NeuTHORS with a gamma monitor placed in the BNCT irradiation room was also done. With this platform, the BNCT information can be immediately and simultaneously monitored by the medical and technical support team or experiment users and operators and thus promote the irradiation quality and control performance of BNCT. The design of NeuTHORS is a good model of a neutron monitoring system that can be utilized on an accelerator-based BNCT facility.

## Data Availability

The data presented in this work are available upon reasonable request from the authors.
